# Anti-*Malassezia*-Specific IgE Antibodies Production in Japanese Patients with Head and Neck Atopic Dermatitis: Relationship between the Level of Specific IgE Antibody and the Colonization Frequency of Cutaneous *Malassezia* Species and Clinical Severity

**DOI:** 10.1155/2011/645670

**Published:** 2011-12-29

**Authors:** Enshi Zhang, Takafumi Tanaka, Mami Tajima, Ryoji Tsuboi, Hiroshi Kato, Akemi Nishikawa, Takashi Sugita

**Affiliations:** ^1^Department of Dermatology, Tokyo Medical University, 6-7-1 Nishishinjuku, Shinjuku, Tokyo 160-0023, Japan; ^2^Department of Microbiology, Meiji Pharmaceutical University, 2-522-1 Noshio, Kiyose, Tokyo 204-8588, Japan; ^3^Department of Immunobiology, Meiji Pharmaceutical University, 2-522-1 Noshio, Kiyose, Tokyo 204-8588, Japan

## Abstract

Atopic dermatitis of the head and neck (HNAD) is recognized as a separate condition. *Malassezia*, the predominant skin microbiota fungus, is considered to exacerbate atopic dermatitis (AD), especially HNAD. In the present study, we investigated the relationships between the levels of specific IgE antibodies, colonization frequency of eight predominant *Malassezia* species, and clinical severity in 61 patients with HNAD (26 mild, 24 moderate, and 11 severe cases). As clinical severity increased, the levels of specific IgE antibodies against eight *Malassezia* species also increased. Species diversity of the *Malassezia* microbiota in scale samples from patients was analyzed by nested PCR using species-specific primers. The clinical severity of HNAD was correlated with the total level of specific IgE antibodies against *Malassezia* species and the number of *Malassezia* species detected.

## 1. Introduction

Atopic dermatitis (AD), which is characterized by dermatitis with pruritus, is a chronic disease that exhibits repeated periods of remission and deterioration. This disease is caused by hypersensitivity to dry skin, a predisposing atopic factor in which IgE antibodies related to the allergy are readily produced, and various environmental factors. Numerous IgE-inducing allergens play a role in the pathogenesis of AD. In fact, specific IgE antibodies against environmental allergens such as mites and various food allergens are detectable in the sera of patients with AD. Since the human body is covered with an enormous number of microorganisms of a plethora of types [1], some may exacerbate the symptoms of AD. *Staphylococcus aureus* is an exacerbating factor in AD, and staphylococcal superantigen-specific IgE is found in the serum of patients with AD, but not healthy individuals [2, 3]. Normally, *S. aureus* does not colonize healthy skin. The skin pH of patients with AD is neutral while that of healthy individuals is weakly acidic, and *S. aureus* does not grow well on healthy skin because it prefers a neutral pH.

With respect to skin fungi, approximately 50 species colonize the skin of patients with AD [4], although the predominant fungus on the skin is the lipophilic yeast *Malassezia*. These microorganisms colonize sebum-rich areas such as the head, face, and neck rather than the limbs or trunk because *Malassezia* requires lipids for growth. During the last two decades, *Malassezia* has been considered to be an exacerbating factor in AD because anti-*Malassezia*-specific IgE is present in the serum of patients with AD, but not healthy individuals [5–7]. In addition, clinical investigations with antifungal agents (ketoconazole or itraconazole) showed improvements in AD symptoms, particularly atopic dermatitis of the head and neck (HNAD) [8–12].

The presence of anti-*Malassezia* IgE has been demonstrated in patients with HNAD [7, 13–15]. The detection frequency of anti-*Malassezia*-specific IgE in serum was higher in patients with HNAD than in those without. Bayrou et al. [7] found IgE against *Malassezia* antigen in 100% of 106 patients with HANAD, but in only 28% of 25 patients who had AD without head and neck involvement. A significant correlation was also observed between the level of *Malassezia*-specific IgE and clinical severity criteria, as reflected by the SCORAD index.

Currently, 14 species are recognized within the genus *Malassezia. *Of them, both *M. globosa* and *M. restricta* were detected in all scale samples of patients with AD when the *Malassezia* microbiota was analyzed by molecular-based culture-independent methods [16]. The colonization level of *M. restricta* was approximately 1.6 times greater than that of *M. globosa* [17]. *M. sympodialis* was the third most predominant species, with a detection rate of 58%. Other species, such as *M. dermatitis*, *M. furfur*, *M. obtusa*, or *M. slooffiae*, were detected in less than 30% of the cases.

In this study, we investigated the relationships between specific IgE and the colonization frequency of the eight most predominant *Malassezia *species and clinical severity levels in patients with HNAD to determine the factors that were correlated with clinical severity.

## 2. Methods

### 2.1. Subjects

Outpatients with HNAD (*n* = 61) at Tokyo Medical University Hospital were enrolled. The study involved patients comprising 26 mild (17 men and 9 women; mean age 34.7 ± 10.5 years; range, 20–63), 24 moderate (16 men and 8 women; mean age 33.2 ± 9.7 years; range, 20–64), and 11 severe (7 men and 4 women; mean age 32.7 ± 10.4 years; range 21–51) cases. AD was diagnosed according to the criteria of Hanifin and Rajka [18]. The study protocol was approved by the Institutional Review Board, and informed consent was obtained from all subjects.

### 2.2. Determination of Anti-*Malassezia* IgE Levels

Antigens from each of eight *Malassezia* species were prepared (*M. dermatitis* JCM 11469, *M. furfur* CBS 1878, *M. globosa* CBS 7966, *M. japonica* CBS 9432, *M. obtusa* CBS 7876, *M. restricta* CBS 7877, *M. sympodialis* CBS 7222, and *M. slooffiae* CBS 7956) according to the method of Kato et al. [19]. IgE levels against these antigens were determined using the AlaSTAT microplate system (Diagnostic Products Corporation, Los Angeles, CA, USA) with slight modifications according to the method of Kato et al. [19]. Briefly, the wells of a microtiter plate were coated with 100 *μ*L of each *Malassezia* antigen in phosphate-buffered saline (PBS). Serum (50 *μ*L) was then added, followed by peroxidase-labeled anti-IgE antibodies. After adding TMB (3,3′,5,5′-tetramethylbenzidine dissolved in hydrogen peroxide) substrate solution, the absorbance at 650 nM was measured at 1 s intervals for 5 min. IgE levels (units/mL) in the samples were calculated using a standard curve, and IgE levels greater than 0.35 U/mL were defined as a positive reaction.

### 2.3. Analysis of *Malassezia* Species Diversity

Scale samples were obtained from lesions by stripping with OpSite, which is a transparent dressing (Smith & Nephew, Hull, UK), and *Malassezia* DNA was extracted directly from the dressing according to the method of Sugita et al. [20]. Briefly, the collected dressing was placed in 1 mL of lysing solution (100 mM Tris–HCl (pH 8.0), 30 mM EDTA (pH 8.0), 0.5% sodium dodecyl sulfate) and incubated at 100°C for 15 min. After deproteinization, DNA was precipitated with ethanol and Ethachinmate (Nippon Gene, Toyama, Japan). The DNA pellet was resuspended in 30 *μ*L of TE buffer (10 mM Tris–HCl (pH 8.0), 1 mM EDTA (pH 8.0)) and stored at −20°C until required. The *Malassezia* species diversity was investigated using nested PCR with species-specific primers, following the method of Sugita et al. [20] and Morishita et al. [21]. Briefly, the *Malassezia* internal transcribed spacer or intergenic spacer region of the rRNA gene was amplified by PCR with *Malassezia* universal primers. The product of this first amplification (1 *μ*L) was used in the nested PCR step with species-specific primers. 

## 3. Results

### 3.1. Anti-*Malassezia*-Specific IgE Production

*Malassezia-*specific IgE antibody levels against eight species in each clinical severity group (mild, moderate, and severe) are shown in Table 1. As clinical severity increased, IgE antibody levels against all eight *Malassezia* species were also increased. In patients with mild AD, the level of specific IgE against *M. restricta* was the highest (1.13 ± 2.50 U/mL), followed by *M. dermatitis* (0.74 ± 1.56 U/mL). Specific IgE for these two species was also present at high levels in patients with moderate AD (7.78 ± 8.84 U/mL for *M. restricta* and 6.13 ± 8.09 U/mL for* M. dermatitis*). In severe patients, with the exceptions of *M. slooffiae* and *M. obtusa*, IgE antibody levels against the remaining six *Malassezia *species were each greater than 15 U/mL.

The detection frequencies of specific IgE antibody are also shown in Table 1; greater than 0.35 U/mL was defined as a positive reaction. The detection frequency was also correlated with the severity of symptoms. Specific IgE antibodies against *M. restricta* and *M. dermatitis* were detected in 42.3% of mild AD cases. Specific IgE antibodies against six *Malassezia* species, with the exceptions of *M. slooffiae* and *M. obtusa*, were detected in greater than 70% of moderate AD cases. Specific IgE antibodies against all eight *Malassezia* species were detected in more than 80% of severe cases.

### 3.2. *Malassezia* Skin Colonization Frequency

*Malassezia* DNA in scale samples was detected by nested PCR using species-specific primers. The colonization frequencies of eight *Malassezia* species among the three clinical severity groups were similar (Table 1). Also, these frequencies were similar to those of eight *Malassezia* in healthy subjects. Both *M. restricta* and *M. globosa* were detected in all samples. The third most predominant species was *M. sympodialis* (detected in 54.5–61.5%). The remaining five species were detected in less than 40% of the cases. A total of two to seven *Malassezia* species were detected from each patient. The average number of species detected in each patient was similar among the three clinical severity groups (3.7 ± 1.6, 3.7 ± 1.6, and 3.5 ± 1.4 species in mild, moderate, and severe AD cases, resp.).

The number of species detected in each severity group was compared with the total IgE antibody levels against *Malassezia* species. As the number of detected species increased, total level IgE antibody levels against *Malassezia* species also increased (Table 2).

## 4. Discussion

In this study, we investigated the relationships between specific IgE levels and the colonization frequency of the eight predominant *Malassezia *species and clinical severity in patients with HNAD to elucidate the factors correlated with clinical severity. Some studies have reported that the frequency of anti-*Malassezia*-specific IgE antibodies in serum was higher in patients with HNAD than in those without. However, the detection frequency of anti-*Malassezia*-specific IgE antibodies in patients with HNAD was different in each report: 100% [14], 68% [23], 55% [24], 55% [15], and 35% [13].

Several factors may explain this variance. Of them, the antigen used might have a significant influence on the frequency of detection. These aforementioned studies had prepared *Malassezia* antigens from *M. furfur* or *M. sympodialis* to detect specific IgE antibodies from patients' sera. Unfortunately, these microorganisms are not the predominant species on the skin of patients with AD. *M. globosa* and *M. restricta *were detected in all patients, while *M. furfur* and *M. sympodialis* were observed in 15.4–29.2% and 54.5–61.5% of the patients, respectively. In addition to detection frequency, colonization levels of *M. globosa* and *M. restricta* accounted for greater than 90% of the entire *Malassezia* microbiota [25]. Therefore, *M. globosa* and *M. restricta *may play a role in exacerbating AD. Based on this result, Kato et al. [19] quantified specific IgE against eight *Malassezia* species, *M. dermatitis*, *M. furfur*, *M. globosa*, *M. obtusa*, *M. pachydermatitis*, *M. slooffiae*, *M. sympodialis*, and *M. restricta*, in sera from patients with AD by enzyme-linked immunosorbent assay (ELISA). The level of specific IgE antibodies for *M. restricta* was greater than that against other *Malassezia* species. Competitive ELISA inhibition tests revealed that *M. restricta* contained species-specific as well as shared antigens.

The colonization level of *Malassezia* in scale samples from patients with AD also differed according to clinical severity. The extent of *Malassezia* colonization between patients with mild and moderate AD was similar. However, colonization in severe patients was two- to fivefold higher than that of mild and moderate patients [26]. In addition, the proportions of the two predominant species, *M*. *globosa* and *M*. *restricta*, differed according to the clinical severity. In patients with mild and moderate AD, *M. restricta* predominated over *M. globosa*, whereas the proportions of *M. globosa* and *M. restricta* were almost identical in patients with severe AD. AD severity had no effect on *Malassezia* species diversity in patients with 3.45–3.71 species colonizing the skin; however, the number of species detected ranged from two to seven of a total of eight. The number of species detected in each case correlated with the total level of specific IgE antibodies against *Malassezia* species in each severity group (Table 2).

Therefore, to elucidate the factors associated with the clinical severity of AD, the relationships between specific IgE levels and colonization levels with the eight most predominant *Malassezia *species should be investigated.

## 5. Conclusion

The clinical severity of HNAD was correlated with total levels of IgE antibodies against *Malassezia* species and the number of *Malassezia* species detected in each case.

## Figures and Tables

**Table 1:** Level and detection frequency of specific IgE antibody and colonization frequency of *Malassezia *species.

Species	Specific IgE antibody value (IU/mL) ^a^			Detection frequency (%) of specific IgE antibody			Colonization frequency (%) of *Malassezia *species			
	Mild	Moderate	Severe	Mild	Moderate	Severe	Mild	Moderate	Severe	HS ^b^
*M. restricta*	1.13 ± 2.50	7.78 ± 8.84	22.74 ± 27.52	42.3	87.5	100	100	100	100	100
*M. globosa*	0.41 ± 0.65	3.24 ± 2.74	16.99 ± 13.24	34.6	83.3	100	100	100	100	100
*M. sympodialis*	0.37 ± 0.45	4.46 ± 4.74	20.74 ± 16.52	30.8	83.3	100	61.5	58.3	54.5	36.7
*M. dermatitis*	0.74 ± 1.56	6.13 ± 8.09	34.47 ± 30.89	42.3	79.2	100	38.5	37.5	27.3	30.0
*M. japonica*	0.26 ± 0.26	1.49 ± 1.70	21.85 ± 20.82	26.9	83.3	90.9	11.5	12.5	18.2	10.0
*M. furfur*	0.28 ± 0.36	2.29 ± 2.89	11.92 ± 12.94	26.9	79.2	100	15.4	29.2	18.2	26.7
*M. slooffiae*	0.13 ± 0.09	0.58 ± 0.60	5.55 ± 9.57	3.8	50.0	81.8	19.2	16.7	9.1	16.7
*M. obtusa*	0.16 ± 0.13	1.15 ± 1.35	4.17 ± 2.92	11.5	62.5	90.9	19.2	16.7	18.2	10.0

^a^, Mean + standard deviation;

^b^, HS: healthy subject [22].

**Table 2:** Relationship between the number of species detected and total IgE antibody levels against *Malassezia *species in each case.

Severity group	Number of species detected and total IgE antibody levels (IU/mL)		
	Less than three species	Four or five species	More than six species
Mild	1.27 + 1.33 (13) ^a^	4.33 + 7.00 (9)	8.62 + 6.40 (4)
Moderate	8.75 + 8.41 (12)	43.44 + 20.27 (8)	49.58 + 27.04 (4)
Severe	114.48 + 89.27 (8)	102.38 (1)	248.20 + 123.74 (2)

^a^Mean + standard deviation. Number of sample examined is shown in parentheses.
